# IGFBP2 promotes immunosuppression associated with its mesenchymal induction and FcγRIIB phosphorylation in glioblastoma

**DOI:** 10.1371/journal.pone.0222999

**Published:** 2019-09-27

**Authors:** Yunmian Liu, Chunyan Song, Faping Shen, Jing Zhang, Sonya Wei Song

**Affiliations:** 1 Center for Brain Disorders Research, Capital Medical University, Beijing Institute for Brain Disorders, Beijing Neurosurgical Institute, Beijing, People's Republic of China; 2 Institute for Cancer Genetics, Irving Cancer Research Center, Columbia University, New York, United States of America; Seoul National University College of Pharmacy, REPUBLIC OF KOREA

## Abstract

Immunotherapy shows a promise for treating glioblastoma (GBM), the most malignant and immunosuppressive glioma. The mesenchymal phenotype of cancer cells was frequently reported to be associated with their induction of immunosuppression within the cancer microenvironment. Overexpressed insulin-like growth factor binding protein 2 (IGFBP2) promotes GBM cell migration and invasion, and contributes to glioma progression and cancer recurrence and poor survival in GBM. However, whether IGFBP2 can induce immunosuppression in GBM was not reported yet. Thus, the study applied a syngeneic mouse GBM model, human GBM samples, and cancer-immune cell co-culture experiments to investigate the effect of IGFBP2 on GBM exposed immune cells and its association with the mesenchymal induction. We found that IGFBP2 promoted the mesenchymal feature of GBM cells. The inhibition of IGFBP2 relieved immunosuppression by increasing CD8^+^ T and CD19^+^ B cells and decreasing CD163^+^ M2 macrophages. Further, the IGFBP2-promoted immunosuppression was associated with its induction of the mesenchymal feature of GBM cells and the inhibitory phosphorylated FcγRIIB of GBM exposed immune cells. Blocking IGFBP2 suppressed tumor growth and improved survival of tumor bearing mice in the mouse GBM model. These findings support the notion that targeting the IGFBP2 may present an effective immunotherapeutic strategy for mesenchymal GBMs.

## Introduction

Glioblastomas (GBMs) are the most common and deadly primary brain tumors and have inevitable local recurrence, thus contributing to the most brain tumor-related mortality in adults. The standard therapy for the disease includes surgery, radiotherapy, chemotherapy, and chemoradiotherapy. Despite the improvements in these therapies, the median survival of the patients with GBMs is well under 2 years and few long-term survivors exist[1]. Increasing evidence has supported the interaction between the immune system and the pathogenesis of glioma[2, 3]. A few prognostic immune signatures related to T cells, natural killer (NK) cells, and microglia/macrophages have been reported in gliomas[4, 5].

In the process of tumor development, the biological process of epithelial-to-mesenchymal transition (EMT) is required for cells to obtain mesenchymal traits; EMT is key for the invasion and metastasis of cancer[6, 7]. Upon recurrence after the standard radiotherapy and chemotherapy GBMs are more likely to transit to the mesenchymal subtype[8]. Mesenchymal GBMs were found to bear predominant immune suppression and frequent pre-existing pro-inflammatory response, and therefore may be immune reactive and particularly amenable to immune therapeutic approaches[9]. This notion is supported by the findings of a retrospective analysis of that GBM patients whose tumors had the mesenchymal signature exhibited improved survival following dendritic cell immunotherapy compared with ones with non-mesenchymal signatures[10]. These findings indicate that EMT of cancer cells contributes to immunosuppression in gliomas.

FcγRIIB is an immune inhibitory receptor that expresses on virtually all immune effector cells except T and NK cells. The activation of FcγRIIB by phosphorylation of its immunoreceptor tyrosine-based inhibitory motif (ITIM) suppresses immune effector cells, leading to immune suppression or tolerance for adaptive and innate immunity[11]. Phosphorylated FcγRIIB recruits phosphatases, such as SHIP, to hydrolyze phosphatidylinositol-3,4,5-trisphosphate (PtdIns(3,4,5)P3) into phosphatidylinositol-4,5-bisphosphate (PtdIns(4,5)P2), which ultimately inhibits the recruitment of pleckstrin homology (PH)-domain containing proteins such as BTK and PLCγ that are required for the activation of downstream kinases for immune responsive action. FcγRIIB activation in GBM-induced immunosuppression was not reported before. A related study showed that FcγRIIB knockout in mice decreased CD39^+^Foxp3^+^Treg cells and M2 macrophages in gliomas[12], consistently supporting its importance in modulating adaptive and innate immunity within the GBM microenvironment. We previously reported that FcγRIIB expression was upregulated in advanced gliomas that correlated with poor survival especially in tumors with mesenchymal (MES) feature and wild type *IDH1* [13], suggesting its role in suppressing immune responses in GBM.

Insulin-like growth factor binding protein 2 (IGFBP2) is commonly overexpressed in GBM, and promotes the migration and invasion of cancer cells[14–17]. IGFBP2 carries Arg-Gly-Asp (RGD) domain that binds to integrins like α5β1 for glioma cell migration, while RGD → RGE mutant (D306E) IGFBP2 could not interact directly with integrin α5 resulting in losing cell mobility[15]. In addition, IGFBP2 activates the nuclear factor-κB pathway to drive EMT and induce invasive characteristics in pancreatic ductal adenocarcinoma cells[18]. The poor prognosis associated with the GBM mesenchymal subtype, and the link between IGFBP2 and key signature genes, such as STAT3[19] and VEGF[20], indicate that IGFBP2 is an important factor in the mesenchymal subtype of GBM[21]. However, whether IGFBP2 participates in promoting immunosuppression by inducing the mesenchymal phenotype in GBM remains unknown. In this study, we investigated the significance of IGFBP2-induced EMT in antitumor immune responses in a GL261 model of glioma.

## Materials and methods

### Cell lines and mice

Mouse GBM cell line GL261 originally from NCI and human GBM cell line LN229 originally from ATCC were cultured in a 37°C, 5% CO2 humidified incubator using 10% FBS-contained RPMI1640 and DMEM/F12, respectively. Six-week-old female C57BL/6J mice were purchased from Vital River (the Chinese distributor of Charles River Laboratory, USA) and habituated to the colony for at least seven days before random assignment to an experimental group. Mice were housed on a 12-hour light/dark cycle under specific pathogen free (SPF) condition with no more than 5 mice per cage and were given water and standard rodent chow ad libitumunder. Animals health and behavior were monitored thrice per week. All experimental procedures were approved by the Animal Care and Use Committee of the Capital Medical University (AEEI-2014-075).

### Cell transfection and treatment

IGFBP2 knockdown with lentiviral shRNA or overexpression of IGFBP2 wildtype (IGFBP2OE) or mutant (IGFBP2mt) with the mutation of its RGD to RGE in GL261 cells was performed according to the manufacturer’s instructions (Genechem Corporation) or generated by plasmid transfection using Lipofectamine 2000 Transfection Reagent (Invitrogen, Thermo Fisher Scientific) under the selection of 400 μg/ml of G418. The pcDNA3.1-IGFBP2 and pcDNA3.1-IGFBP2mt expression vectors were constructed by standard subcloning procedures. The mutation of RGD to RGE was generated by using the QuikChange site-directed mutagenesis kit (Stratagene). For anti-IGFBP2 antibody treatment, 2.0 μg/ml of a rabbit anti-IGFBP2 monoclonal antibody (anti-IGFBP2mAb) (bs-1108R, Bioss) or isotype IgG control (bs-0295P, Bioss) was added to GL261 culture for 3 days and collected for western blotting. The interfering RNA sequences for IGFBP2 knockdown in the lentiviral vector are listed in Table 1.

**Table 1:** The interfering RNA sequences used for IGFBP2 knockdown.

Group	Interference RNA sequence
Control Group (sh-Ctrl)	TTCTCCGAACGTGTCACGT
IGFBP2 knockdown group 1 (sh1-IGFBP2)	ATGCCCAAAGTGTGCAGTA
IGFBP2 knockdown group 2 (sh2-IGFBP2)	AGTGCAAGATGTCTCTGAA

### Cell co-culture of mouse SPCs and GBM cells

Mouse spleen from a six week-old female C57BL/6 mouse was dissected and dispersed into single-cell suspensions that were then filtered and centrifuged through a 30/70% Percoll gradient at 7800g for 30 minutes. Mouse spleen lymphocytes (SPCs) at the interface were collected, washed, and used for cell co-culture experiments. With the ratio of 10:1 of SPCs versus cancer cells, SPCs were co-cultured with GL261 cells pretreated with 10 μM mitomycin C for 45 minutes for indicated times. To block extracellular IGFBP2, 2.0 μg/ml of a rabbit anti-mIGFBP2 monoclonal antibody (anti-IGFBP2mAb) (bs-1108R, Bioss) or isotype IgG control (bs-0295P, Bioss) was added to GL261 culture 30 minutes before co-cultured with SPCs and then added at a 3-day interval. Six days later unless indicated, SPCs were collected and analyzed for indicated protein markers by fluorescence-activated cell sorting (FACS).

### Mouse GBM model and treatment

Orthotopic implantation of cancer cells was performed as previously described[14]. Briefly, GL261 cells (5×10^4^ cells in 3μl) were intracranially injected into mice (2.1 mm lateral to the bregma and 2.7 mm below the surface of the brain) under anesthesia using a stereotactic instrument. T2 image was scanned with magnetic resonance imaging (MRI) equipment (BRUKER, PharmaScan 7T). For antibody treatment, the mice were randomly assigned to a control or treatment group (15 mice per group) in which 5 mice were used for the analysis of tumor growth and mouse survival and 10 mice were euthanized for the analysis of IHC and Flow cytometry at the fourth week after the antibody treatment. After seven days of the cell injection, the mice were intraperitoneally treated with 100 μg/kg of a rabbit anti-IGFBP2 antibody or isotype IgG (bs-0295P, Bioss) twice per week until the complete death of control mice. Tumor volume is calculated by multiplying the sum of the areas in a continuous T2 image of the tumor by the spacing between the layers. The survival time was recorded from the injection day until the day of death, or showing severely neurological symptoms, or losing 15–20 percent of body weight quickly. Once animals reached endpoint criteria, they were euthanized immediately. In the study mice were anesthetized with isoflurane and euthanized via a bilateral pneumothorax.

### Tissue microarrays

Human GBM tissue microarrays purchased from Alenabio Corporation including 35 primary GBMs, two adjacent GBM tissues, and three normal brains were stained with anti-IGFBP2 (bs-1108R) or anti-p-FcγRIIB (bs-6031R) antibodies from Bioss by following standard IHC staining procedures. Signal intensity was scanned using Leica Aperio AT2 instrument and analyzed with Aperioe Slide Manager Software.

### Immunohistochemistry staining (IHC)

Four weeks after the antibody treatment, brains were dissected, paraffin-embedded, and sectioned into 5.0 μm slices for IHC staining. Briefly, after antigen retrieval in 0.1 M citrate buffer (PH 6.0), sections were incubated overnight at 4°C with primary antibodies and then stained with horseradish peroxidase-conjugated secondary antibody (PV-6000, Polymer detection system) followed by DAB detection system (DAB-2031, MaxVision) according to the manufacturer’s instructions. Primary monoclonal antibodies used for staining included anti-IGFBP2 (bs-1108R) and anti-p-FcγRIIB (bs-6031R) from Bioss and anti-CD8 (ab209775), anti-p-CD19(Y531) (ab203615), and anti-CD163 (ab182422) antibodies from Abcam. After staining, the slides were scanned (original magnification:400X,100X) using Leica Aperio AT2 instrument. Cytoplasmic v2 algorithm was chosen for antibody positivity in cytoplasm. Positive cells were counted using Image J software in 5 separate regions randomly inside the tumor (Intratumoral) and at the tumor edge (Tumor Edge) on each slide. The total number of labeled cells were graded as +1, +2, and +3 for intensity, and normalized to cells/mm^2^ by taking the cell count and dividing by the area of the region (mm^2^) then averaging the 5 separate regions. The automated-derived counts were used to calculate cells/mm^3^ by multiplying the cell count/mm^2^ by 250 (250 slices per biopsy when each sectioned slice is made of 5 μm sections in 1mm thickness) and dividing by correction factor based on the average number of sections in which an immunocyte appears in 5 μm thick sections. For statistical analyses, averages of at least three sections from 3 biopsies were utilized (Student t-test)[22].

### Flow cytometry

Tumor-infiltrating immune cells from mouse brains were isolated according to previously published protocol[23] after 4 weeks of the antibody treatment. Briefly, a whole tumor-bearing mouse brain except cerebellum was gently homogenized with a grinder and filtered to obtain single cell populations, and then centrifuged through a 30/70% Percoll gradient at 7800 g for 30 minutes. The leukocyte cells at the interface were collected, washed, and analyzed by FACS using indicated antibodies. Cell surface staining was performed with APC, PE, or FITC-labeled anti-CD4 (553051, BD Biosciences), anti-CD8 (553030, BD Biosciences), anti-CD19 (12-0193-82, ebioscience, Thermo Fisher Scientific), anti-CD163 (12-1631-80, ebioscience, Thermo Fisher Scientific) or appropriate isotype-matched control antibodies. For anti-F4/80 (MF48000, Invitrogen, Thermo Fisher Scientific) mAb, Alexa Fluor 647-labeled secondary antibodies (ab150155, abcam) were applied. For intracellular staining of p-FcγRIIB, cells were then treated with fixation/permeabilization buffer (562574, BD Pharmingen, BD Biosciences) and incubated with anti-p-FcγRIIB (bs-6031R, Bioss) antibody followed with Alexa Fluor® 405-labeled secondary antibody according to the manufacturer’s instructions. Stained cells were analyzed with Amnis Image Stream Mark II (Millipore Corporation) and the data were presented using IDEAS software. For in vitro cancer-immune cell co-culture experiments, after cultured with GL261 cells, SPCs were collected and washed with PBS for FACS.

### ELISA

The peripheral blood was collected from an eye area of an anesthetized mouse after five week-cell inoculation and centrifuged to obtain the serum. Serum TGFβ1 (MB100B, R&D System) and IGFBP2 (EMIGFBP2, Thermo Fisher Scientific, with the minimum detectable level of 0.823 ng/ml) were measured using ELISA kits according to the manufacturer’s instructions.

### Western blot

Western blot analysis was performed by using standard protocols. Cell lysate was extracted using RIPA buffer (Cell Signaling) supplemented with 1mM phenylmethylsulfonyl fluoride (PMSF) just before use. Primary antibodies used were: IGFBP2 (ab13649, abcam), fibronectin (610077, BD), CD44 (ab157107, abcam), vimentin (5741, Cell Signaling), and β-actin (A5316, Sigma Aldrich); the goat-anti-mouse (SA00001-1) or rabbit (SA00001-2) second antibodies were bought from Proteinteck Company.

### Glioma database and statistical analysis

Raw read counts of genes from HTseq were obtained from the The Cancer Genome Atlas (TCGA) for 667 primary gliomas including 216 grade II and 237 grade III gliomas and 153 GBMs and normalized by EdgeR package and log transformed for further analysis as indicated in the text. MES signature genes (S1 Table)[24] were used to calculate the enrichment score of each glioma sample. Pearson correlation coefficients were calculated in regard to IGFBP2, p-FcγRIIB, and MES signature as indicated. Significant differences were calculated by using an unpaired Welch’s t test. Survival time was assessed with Kaplan-Meier plotting and log-rank test. The data analysis was performed using with GraphPad Prism 6, R and SPSS 16.0. P < 0.05 was considered statistically significant.

## Results

### IGFBP2 promotes the mesenchymal feature of glioma cells

**Fig 1:**
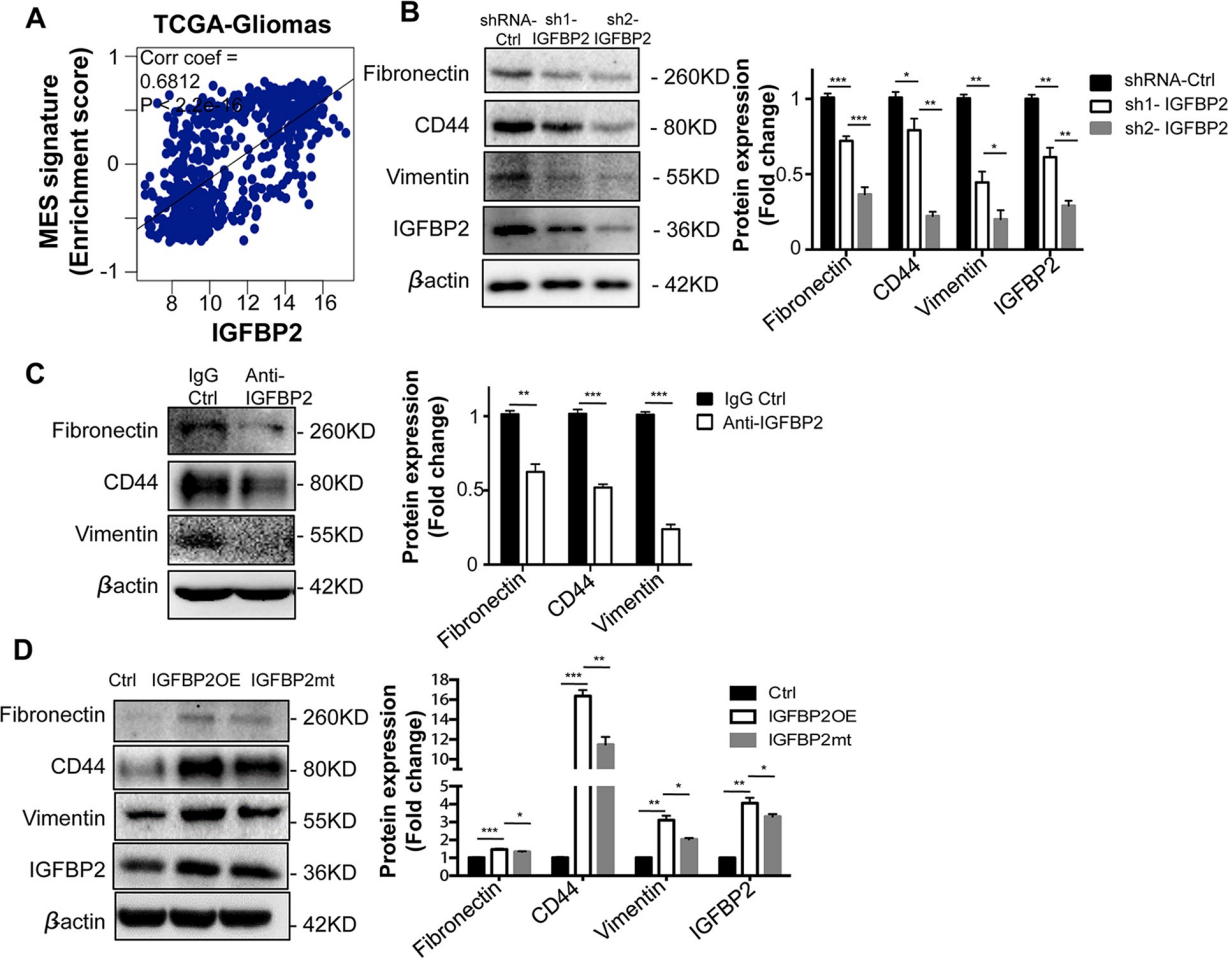
IGFBP2 promoted the mesenchymal feature of glioma cells. (A) An association of the enrichment score of the MES signature with the expression of IGFBP2 using the TCGA-gliomas database. R = Pearson’s correlation coefficient. (B-C) Effect of IGFBP2 knockdown with shRNA (IGFBP2KD) (B) or an anti-IGFBP2 antibody (anti-IGFBP2) (C) on mesenchymal marker protein levels in mouse GBM GL261 cells. (D) Effect of overexpressed IGFBP2 wildtype (IGFBP2OE) or mutant (IGFBP2mt) with the change of RGD to RGE on mesenchymal marker protein levels. The experiments were repeated at least three times. A statistical significance was calculated using an unpaired Welch’s t test. * P<0.05, ** P<0.01, *** P<0.001.

Many studies showed that IGFBP2 promoted GBM cell migration and invasion[14, 15]. To determine a direct association of IGFBP2 with the mesenchymal feature of gliomas, we first analyzed the expression association of IGFBP2 with the mesenchymal signature of gliomas by using the TCGA-human glioma gene expression database. The enrichment score of the mesenchymal signature of gliomas was significantly positively correlated with IGFBP2 expression in human gliomas (R = 0.6812, p < 2.2e-16) (Fig 1A). We then used two shRNA-IGFBP2 clones (IGFBP2KD) or an anti-IGFBP2 antibody (anti-IGFBP2) directly to inhibit IGFBP2 in mouse GBM cells (GL261 cells) and found that mesenchymal marker proteins [25, 26] fibronectin, CD44, and vimentin were correspondingly markedly decreased compared to the control (Fig 1B and 1C, * p < 0.05, ** p < 0.01, *** p < 0.001). In contrast, overexpressed IGFBP2 (IGFBP2OE) increased the expression of those marker proteins (Fig 1D, ** p < 0.01, *** p < 0.001), but IGFBP2 mutant (IGFBP2mt) reduced the ability of IGFBP2 to increase those marker protein levels (Fig 1D, * p < 0.05, ** p < 0.01), suggesting that the IGFBP2-promoted mesenchymal feature be partially regulated through its binding to integrin.

### Inhibition of IGFBP2 increases CD8^+^ T and p-CD19^+^ B cells and decreases CD163^+^ M2 macrophages

**Fig 2:**
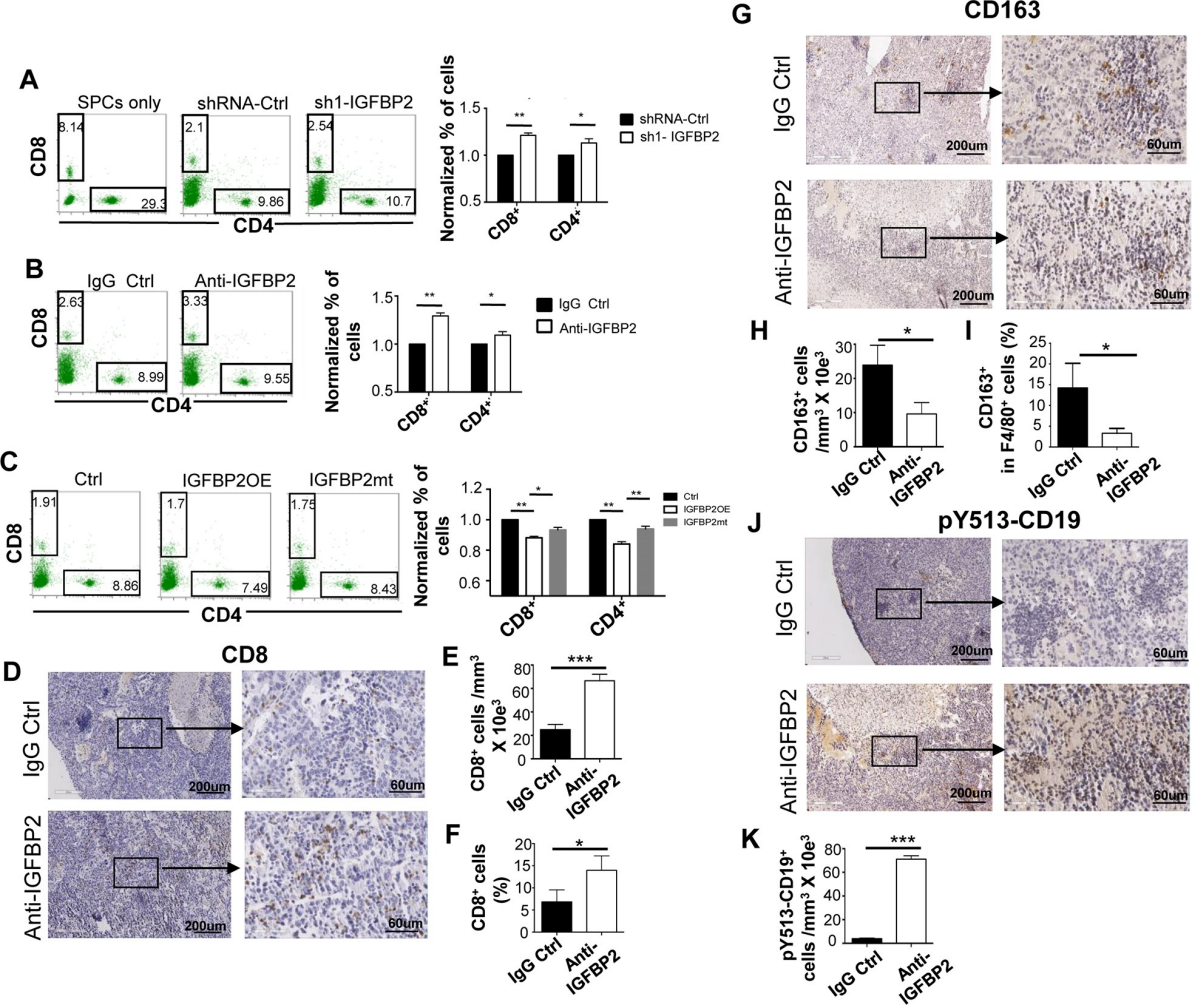
Effect of IGFBP2 inhibition on the number of T, B cells and M2 macrophages. (A-C) The mouse splenocytes (SPCs) were co-cultured with GL261 cells for 6 days and then collected to analyze the number of CD8^+^ and CD4^+^ T cells by fluorescence activated cell sorting (FACS) under the setting of IGFBP2KD (A), anti-IGFBP2 (B) or overexpressed IGFBP2 and its mutant (C). The experiments were repeated at least three times. Representative IHC images of CD8^+^ T cells (D-E), CD163^+^ M2 macrophages (G-H), and pY513-CD19^+^ cells (J-K) in tumor-bearing brains of mice treated with anti-IGFBP2 or IgG. 100X (left), 400X (right). N = 3. (F, I) The percentage of tumor infiltrating CD8^+^ T cells (F) and CD163^+^ M2 macrophages in F4/80^+^ macrophages (I) analyzed by FACS after anti-IGFBP2 or IgG treatment. N = 5. The data are mean ± SD. A statistical significance was calculated by an unpaired Welch’s t test. * P<0.05, ** p < 0.01, *** p < 0.001.

As the mesenchymal traits are tightly related to immunosuppression in cancer, we then examined the effect of IGFBP2 on immune cells exposed to GBM cells by using the GL261-SPCs co-culture experiments and the mouse GBM model. The number of CD8^+^ and CD4^+^ T cells after co-cultured with IGFBP2KD cells for 6 days was significantly increased compared to the control (Fig 2A, * p < 0.05, ** p < 0.01). Anti-IGFBP2 generated the similar results (Fig 2B, * p < 0.05, ** p < 0.01). Conversely, IGFBP2OE significantly decreased the number of CD4^+^ and CD8^+^ T cells compared to the control (Fig 2C, * p < 0.05, ** p < 0.01). IGFBP2mt had a less ability to decrease the number of CD8^+^ and CD4^+^ T cells compared to IGFBP2OE (Fig 2C, * p < 0.05, ** p < 0.01), consistent with its decreased mesenchymal induction.

As the serum protein level of IGFBP2 was significantly increased in the orthotopic GL261 tumor bearing mice compared to the no tumor mice (S1A Fig, ** p < 0.01), we treated the mice with anti-IGFBP2 intraperitoneally after 7 days injection of tumor cells until the complete death of the control mice. The immunohistochemical staining (IHC) showed an increased infiltration of CD8^+^ T cells in anti-IGFBP2 treated tumors compared to IgG treated tumors (Fig 2D–2E, *** p < 0.001). Anti-IGFBP2 effectively increased the tumoral accumulation of CD8^+^ T cells detected by FACS (Fig 2F, * p < 0.05).

Macrophages are the major components in human GBM tissues (commonly named tumor associated macrophages, TAMs), in which immunosuppressive M2 TAMs is a major subtype, having a profound impact in the survival of GBM patients[1, 27, 28]. We thus examined the presence of CD163^+^(a marker of M2 subtype) TAMs in the GL261 tumors. Anti-IGFBP2 significantly decreased the number of infiltrated CD163^+^M2 TAMs (Fig 2G–2I, * p < 0.05).

Our previous study showed that a B cell-associated predictive gene signature could identify high risk patients with high-grade gliomas (grade III and GBM) for poor survival and suitable for chemotherapy in addition to radiotherapy, indicating the importance of B cell presence in gliomas[13]. We observed that anti-IGFBP2 tumors had a marked increase of pY513-CD19^+^ B cells (Fig 2J). The phosphorylation of CD19-Y513 indicates the activation of CD19[29]. In addition, we also detected decreased serum levels of immunosuppressive TGF-β1 in anti-IGFBP2 treated mice (S1B Fig, * p < 0.05).

### IGFBP2 promotes the inhibitory phosphorylated FcγRIIB on GBM exposed immune cells associated with its mesenchymal induction

Our previous study demonstrated that the expression of the immune inhibitory gene FcγRIIB was a risk factor for poor survival and correlated with the immunosuppressive microenvironment in high-grade gliomas[13].We thus analyzed the association of FcγRIIB expression with the mesenchymal signature and IGFBP2 expression in gliomas.

**Fig 3:**
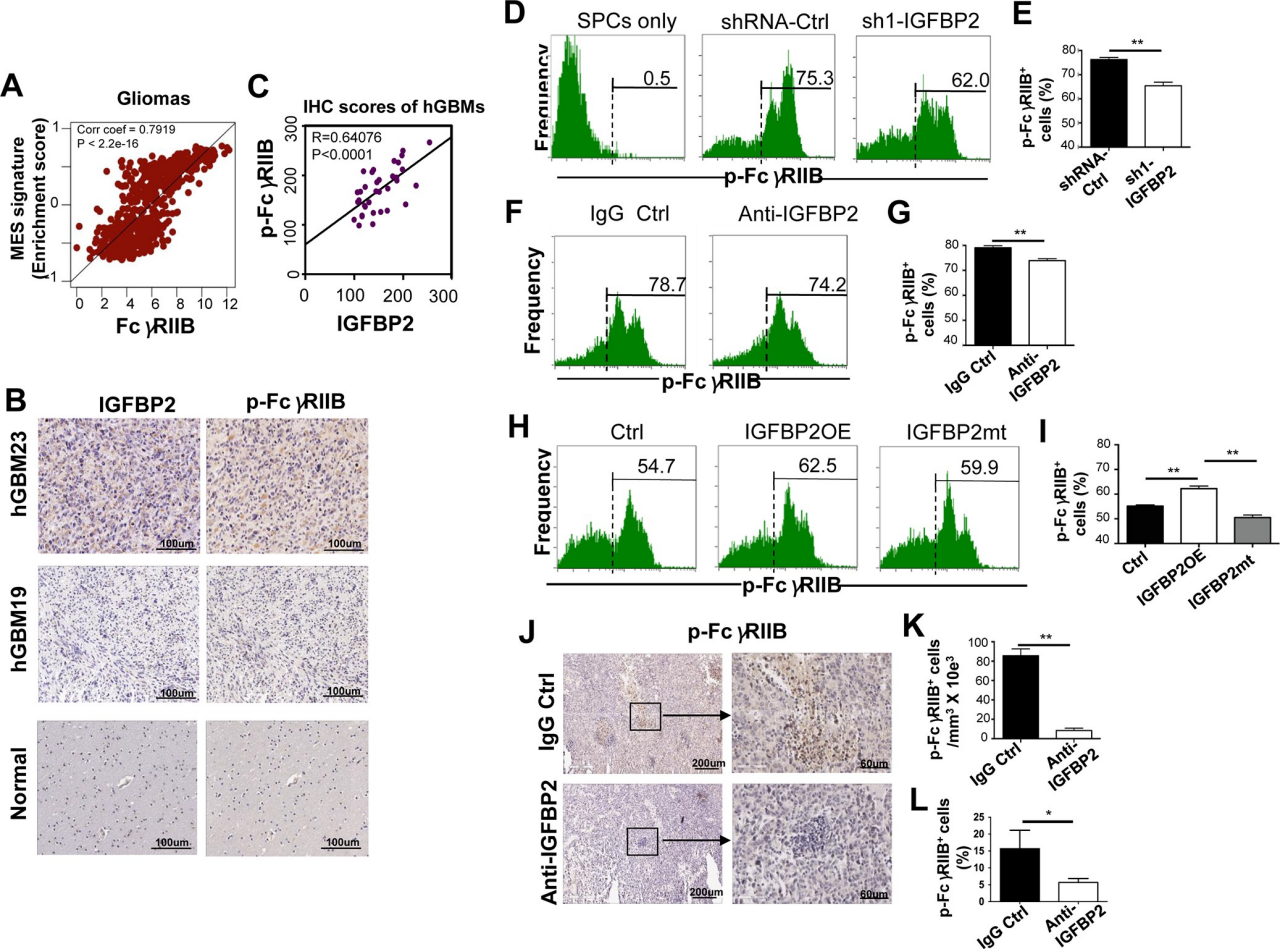
IGFBP2 induction of the phosphorylation of FcγRIIB on GBM exposed immune cells. **(**A) An association of the enrichment score of the MES signature with FcγRIIB expression analyzed by using the TCGA-human glioma database. R = Pearson’s correlation coefficient. (B) Representative IHC images of IGFBP2 protein and phosphorylated FcγRIIB (p-FcγRIIB) levels in human GBMs (hGBMs) or normal brains stained in tissue microarrays. (C) An association of IGFBP2 protein and p-FcγRIIB levels in human GBMs. R, the Pearson correlation coefficient. (D-I), The percentage of p-FcγRIIB^+^ cells in the SPCs after co-cultured with GL261 cells treated with sh1RNA-IGFBP2 (D-E), anti-IGFBP2 (F-G) or overexpressing IGFBP2 or IGFBP2mt (H-I) analyzed by FACS. (J-K), Representative IHC images of p-FcγRIIB staining in tumor-bearing brains treated with anti-IGFBP2 or IgG. N = 3. (L) The percentage of p-FcγRIIB^+^ cells in infiltrating immune cells in GL261 tumors analyzed by FACS. N = 5. The data are mean ± SD. A statistical significance was computed by an unpaired Welch’s t test. * P < 0.05, ** p < 0.01.

FcγRIIB expression was significantly correlated with the enrichment score of the mesenchymal signature (R = 0.7919, p < 2.2e-16) (Fig 3A) and moderately with IGFBP2 expression (R = 0.22, p < 0.012) in gliomas. As the phosphorylation of FcγRIIB activates its inhibitory function[11], we then assessed the association of phosphorylated FcγRIIB (p-FcγRIIB) with IGFBP2 expression in human GBMs by using tissue microarrays. In the 35 human GBMs, 62.85% (22/35) of GBMs was p-FcγRIIB—positive and 77.14% (27/35) IGFBP2—positive, but normal brains exhibited a weak or negative p-FcγRIIB and IGFBP2 (Fig 3B). Phosphorylated FcγRIIB was significantly correlated with IGFBP2 expression in human GBMs (R = 0.6417, p < 0.0001) (Fig 3C).

We then determined whether GBM-expressed IGFBP2 directly impacted in p-FcγRIIB on immune cells when exposed to GBM cells with cell co-culture and the mouse GBM model experiments. IGFBP2KD markedly reduced the number of p-FcγRIIB^+^ cells in SPCs co-cultured with GL261 cells compared to the control (Fig 3D–3E, ** p < 0.01). No p-FcγRIIB^+^ cells were detected in SPCs only (Fig 3D). Similarly, Anti-IGFBP2 also decreased p-FcγRIIB^+^ cells in SPCs (Fig 3F and 3G, ** p < 0.01). Conversely, IGFBP2OE increased p-FcγRIIB^+^ cells in SPCs, but IGFBP2mt had less ability to increase p-FcγRIIB^+^ cells in SPCs relative to IGFBP2OE (Fig 3H and 3I, ** p < 0.01). Concordantly, anti-IGFBP2 tumors in the mouse brains showed a markedly decreased p-FcγRIIB^+^ cells compared to control tumors (Fig 3J–3L, * p < 0.05, ** p < 0.01).

### IGFBP2 increases p-FcγRIIB on B cells and macrophages

**Fig 4:**
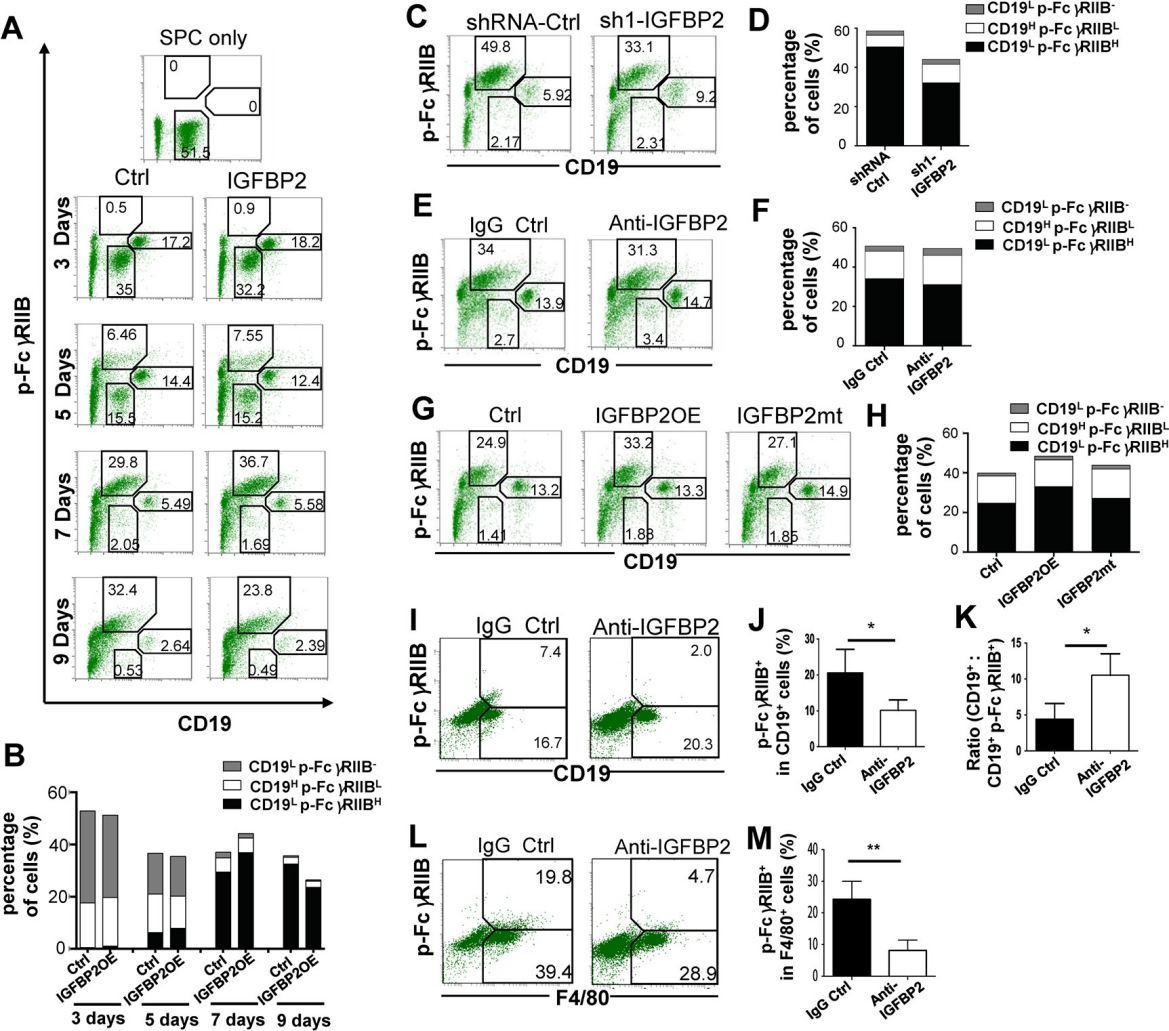
Effect of IGFBP2 on the number of p-FcγRIIB^+^ cells in B cells and macrophages. (A-B) The time course induction of FcγRIIB phosphorylation on CD19^+^ B cells in SPCs co-cultured with IGFBP2OE GL261 or control cells. The percentage of CD19^L^ p-FcγRIIB^H^, CD19^L^ p-FcγRIIB^-^, and CD19^H^ p-FcγRIIB^L^ B cell subsets was analyzed by FACS. L is low, H is high. (C-H) The percentage of p-FcγRIIB^+^ cells in CD19^+^ B cells after co-cultured with GL261 cells treated with shRNA (C-D), anti-IGFBP2 (E-F) or overexpressing IGFBP2 or its mutant (G-H). (I-J) The percentage of CD19^+^ p-FcγRIIB^+^ B cells in infiltrating immune cells (I) and p-FcγRIIB^+^ cells in CD19^+^ B cells in anti-IGFBP2 or IgG tumors analyzed by FACS(J). (K) The ratio of CD19^+^ B cells versus CD19^+^ p-FcγRIIB^+^ B cells. (L-M) The percentage of F4/80^+^p-FcγRIIB^+^ macrophages in infiltrating immune cells (L) and p-FcγRIIB^+^ cells in F4/80^+^ macrophages (M) in anti-IGFBP2 or IgG tumors analyzed by FACS. N = 5. The data are mean ± SD. A statistical significance was calculated by an unpaired Welch's t test. * P < 0.05, ** P < 0.01.

Next, we dissected the impact of IGFBP2 in p-FcγRIIB on B cells because FcγRIIB is the only inhibitory receptor on B cells and the FcγRIIB phosphorylation is well known to inhibit B cell proliferation, activation, and function[11]. The time course induction of p-FcγRIIB on B cells showed that IGFBP2OE increased the accumulation of CD19^L^p-FcγRIIB^H^ subpopulation and meanwhile, decreased the number of CD19^L^p-FcγRIIB^-^ and CD19^H^p-FcγRIIB^L^ subpopulations more rapidly, eventually leading to a more decreased number of CD19^+^ B cells after extended culture with GL261 cells (Fig 4A and 4B, p values were shown in Table 2). In contrast, IGFBP2KD or anti-IGFBP2 decreased p-FcγRIIB^+^ cells in CD19^+^ B cells relative to the control (Fig 4C–4F, p values were shown in Table 3). Consistent with above findings, IGFBP2mt had a decreased ability to promote FcγRIIB phosphorylation on B cells relative to IGFBP2OE (Fig 4G and 4H, p values were shown in Table 3). In the mouse GBM, anti-IGFBP2 also significantly decreased CD19^+^p-FcγRIIB^+^ B cells (Fig 4I and 4J, * p < 0.05) and increased the number of CD19^+^ B cells corresponding to p-FcγRIIB^+^ B cells (Fig 4K, * p < 0.05).

**Table 2:** P values in Fig 4B.

	3 days	5 days	7 days	9days
CD19^L^p-FcγRIIB^-^	**	Ns	**	Ns
CD19^H^ p-FcγRIIB	*	**	Ns	Ns
CD19^L^p-FcγRIIB^H^	*	**	**	**

* p < 0.05

** p < 0.01

*** p < 0.001

**Table 3:** P values in Fig 4C–4H.

	Ctrl Vs sh1-IGFBP2	Ctrl Vs anti-IGFBP2	Ctrl Vs IGFBP2OE	IGFBP2OE Vs IGFBP2mt
CD19^L^pFcγRIIB^-^	Ns	*	*	Ns
CD19^H^ p-FcγRIIB	**	*	Ns	Ns
CD19^L^pFcγRIIB^H^	**	*	***	**

* p < 0.05

** p < 0.01

*** p < 0.001

As macrophages are important in GBM immunology, on which FcγRIIB is present, we also examined the impact of IGFBP2 in FcγRIIB phosphorylation on TAMs. We found that anti-IGFBP2 significantly decreased p-FcγRIIB^+^ cells in F4/80^+^ TAMs (Fig 4L and 4M, ** p < 0.01).

### Blocking IGFBP2 suppresses tumor growth and improves survival in the mouse GBM model

**Fig 5:**
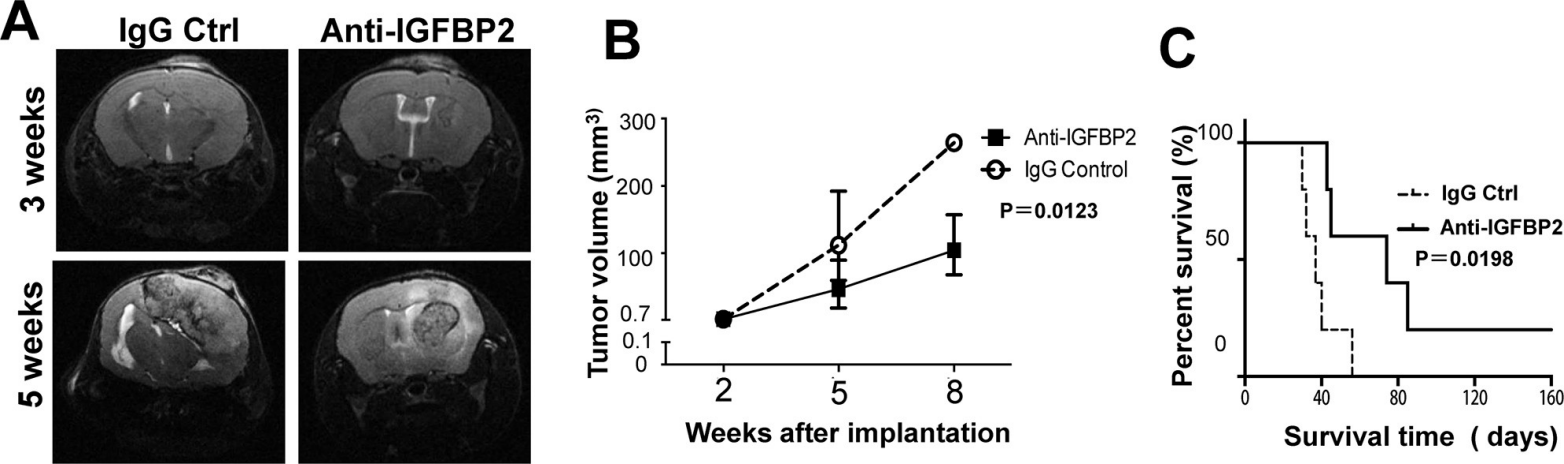
IGFBP2 blockade decreased tumor growth and improved survival in the mouse GBM model. **(**A) Representative T2-weighted MRI of GL261-bearing brains of mice treated with anti-IGFBP2 or IgG after 3 and 5 weeks of GL261cell injection. (B) The tumor growth curve in anti-IGFBP2 and IgG groups. (C) The Kaplan-Meier survival plot of GL261-bearing mice treated with anti-IGFBP2 or IgG. Log-rank test was used to compare the difference of the median survival time between the two groups. N = 5.

Further, we assessed a therapeutic effect of IGFBP2 blockade in the mouse GBM model. Anti-IGFBP2 decreased the tumor volume of GBM in mice brains (p = 0.0123) (Fig 5A and 5B) and improved the median survival of tumor-bearing mice compared to IgG control (median survival time: 39.0 days versus 93.4 days, p = 0.0198) (Fig 5C), suggesting that blocking IGFBP2 may have therapeutic effects on human GBM.

## Discussion

Previous studies showed that amino terminus-derived epitopes of IGFBP2 elicited T cell immunity by production of IFN-γ through T helper 1 (Th1) cells, but which is abrogated by its carboxyl terminus-derived epitopes through inducing Th2-producing IL-10[30–32]. Our study is the first showing that IGFBP2 promotes the mesenchymal feature of GBM cells and induces immunosuppression within the GBM microenvironment. Recently, we have reported that IGFBP2 was overexpressed in the four GBM molecular subtypes (classical, neural, proneural, and mesenchymal subtypes), but its upregulated expression of cell proliferation genes found in neural stem cells was extremely correlated with its overexpression only in non-mesenchymal GBM cells, but not in mesenchymal ones[33]. This is completely consistent with these findings of that overexpressed IGFBP2 in mesenchymal GBMs promoted their mesenchymal phenotype and contributed to immunosuppression in mesenchymal GBMs. Altogether, our studies have further confirmed that IGFBP2 indeed plays different and critical functions in non-mesenchymal and mesenchymal GBM subtypes through its distinct molecular mechanisms.

The precise molecular mechanisms underlying mesenchyme-induced immunosuppression are still actively under investigation. Several mechanisms are currently proposed to be utilized by cancer cells, including dysregulated expression of immunomodulatory genes and alterations in cytokine and chemokine secretion patterns, which may trigger a cascade of events such as the recruitment of immunosuppressive cells to the cancer microenvironment and reprogramming pre-existing immune cells to immunosuppressive ones, ultimately culminating in immunosuppression[4].

In the study, we demonstrate that IGFBP2 promotes the phosphorylation of FcγRIIB on cancer exposed immune cells, which is associated with its mesenchymal induction. FcγRIIB activation in GBM-induced immunosuppression was not reported before. A related study showed that FcγRIIB knockout in mice decreased CD39^+^Foxp3^+^Treg cells and M2 macrophages in gliomas[12], consistently supporting its importance in modulating adaptive and innate immunity within the GBM microenvironment.

B cell-mediated immune responses are barely studied in cancer immunology including gliomas. Recently, some studies have reported that the high density of B cells in tumors alone or in combination with the number of activated T cells provides a significant survival advantage, indicating that infiltrating B cells have an anti-tumor immune response[34–37]. We show that FcγRIIB-ITIM phosphorylation on B cells expedites B cell reduction and anti-IGFBP2 effectively recovers the B cell population in mouse GBMs. Possibly, our results provide an approach to remove the suppression on B cells and restore their humoral immune response and antigen presenting function for T cell activation within the cancer microenvironment.

It was reported that FcγRIIB expression on macrophages modulated their phagocytic and cytotoxic potential *in vivo*[38]. This study shows that the inhibition of IGFBP2 simultaneously decreases p-FcγRIIB^+^ macrophages and CD163^+^ M2 macrophages in mouse GBMs. The TCGA-glioma data analysis also shows a strong expression correlation of FcγRIIB and CD163 (R = 0.82, p < 2.2e-16). It is likely that IGFBP2-promoted FcγRIIB phosphorylation on TAMs may help the transition of M1 TAMs to M2 TAMs, which deserves a further investigation.

Taken together, as the recruitment of innate and adaptive immunity to initiate a more integrated immune response to cancer is the best way to immunotherapy, targeting the IGFBP2-FcγRIIB pathway may enhance the susceptibility of mesenchymal GBMs to various immunotherapeutic regimens.

## Supporting information

S1 Fig Serum proteins level. (A)Serum IGFBP2 protein levels in no tumor or tumor bearing mice. (B) Serum TGF-β1 protein levels of no tumor mice or tumor-bearing mice treated with IgG or anti-IGFBP2. A statistical significance was computed by an unpaired Welch’s t test. N = 3–5, * *P<0.01, *P<0.05.
(DOCX)

S1 Table Mesenchymal signature genes for statistical ananlysis in the TCGA datebase. (DOCX)
