# Supplementary material for: IGFBP2 promotes immunosuppression associated with its mesenchymal induction and FcγRIIB phosphorylation in glioblastoma
Source: PLoS One. 2019 Sep 27;14(9):e0222999. doi: 10.1371/journal.pone.0222999 (PMC6764691; doi:10.1371/journal.pone.0222999)
Supplement: S1 Fig — (A)Serum IGFBP2 protein levels in no tumor or tumor bearing mice. (B) Serum TGF-β1 protein levels of no tumor mice or tumor-bearing mice treated with IgG or anti-IGFBP2. A statistical significance was computed by an unpaired Welch’s t test. N = 3–5, * *P<0.01, *P<0.05. (DOCX) [file pone.0222999.s001.docx]

**
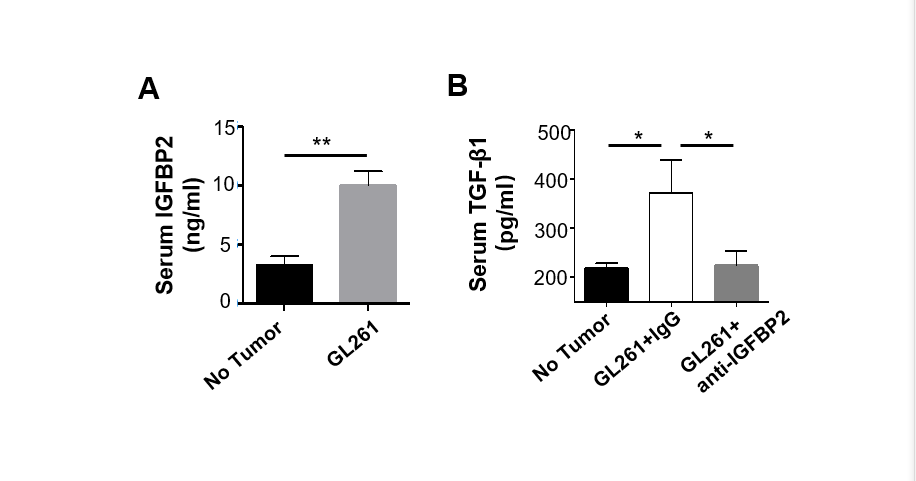
**

**Supporting information**

**S1 Fig. Serum proteins level.** (A)Serum IGFBP2 protein levels in no tumor or tumor bearing mice. (B) Serum TGF-β1 protein levels of no tumor mice or tumor-bearing mice treated with IgG or anti-IGFBP2. A statistical significance was computed by an unpaired Welch’s t test. N=3-5, * *P<0.01, *P<0.05.

**Supplementary Table 1:**

| **Mesenchymal Signature Genes** |  |  |  |  |
| --- | --- | --- | --- | --- |
| ACPP | CTSZ | LCP2 | PTRF | THBD |
| ACSL1 | CYBRD1 | LGALS1 | PYGL | THBS1 |
| ADAM12 | DAB2 | LGALS3 | RAB11FIP1 | TIMP1 |
| AIM1 | DCBLD2 | LHFPL2 | RAB27A | TLR2 |
| ALDH3B1 | DOK3 | LILRB2 | RAB32 | TLR4 |
| ALOX5 | DSC2 | LILRB3 | RABGAP1L | TMBIM1 |
| AMPD3 | DSE | LOX | RAC2 | TNFAIP3 |
| ANXA1 | EFEMP2 | LRRFIP1 | RBKS | TNFAIP8 |
| ANXA2 | EHD2 | LTBP1 | RBMS1 | TNFRSF11A |
| ANXA4 | ELF4 | LTBP2 | RELB | TNFRSF1A |
| ARHGAP29 | EMP3 | LY75 | RHOG | TNFRSF1B |
| ARPC1B | ENG | LY96 | RRAS | TRADD |
| ARSJ | FCGR2A | MAFB | RUNX2 | TRIM22 |
| ASL | FCGR2B | MAN1A1 | S100A11 | TRIM38 |
| BATF | FES | MAN2A1 | S100A13 | TRPM2 |
| BDKRB2 | FHL2 | MAN2B1 | S100A4 | UAP1 |
| BLVRB | FHOD1 | MAPK13 | SAT1 | UCP2 |
| BNC2 | FMNL1 | MFSD1 | SCPEP1 | VAMP5 |
| C1orf38 | FNDC3B | MGAT1 | SEC24D | VDR |
| C1orf54 | FOLR2 | MGST2 | SERPINA1 | WIPF1 |
| C5AR1 | FURIN | MRC2 | SERPINE1 | WWTR1 |
| CASP1 | FXYD5 | MS4A4A | SFT2D2 | YAP1 |
| CASP4 | GCNT1 | MSR1 | SH2B3 | ZNF217 |
| CASP5 | GLT25D1 | MVP | SHC1 | COL8A2 |
| CASP8 | GNA15 | MYH9 | SIGLEC7 | COPZ2 |
| CAST | GRN | MYO1F | SIGLEC9 | CSTA |
| CCDC109B | HEXA | NCF2 | SLAMF8 | CTSB |
| CCR5 | HEXB | NCF4 | SLC10A3 | CTSC |
| CD14 | HFE | NOD2 | SLC11A1 | KYNU |
| CD2AP | HK3 | NPC2 | SLC16A3 | LAIR1 |
| CD4 | ICAM3 | NRP1 | SP100 | LAMB1 |
| CDCP1 | IFI30 | P4HA2 | SQRDL | LAPTM5 |
| CEBPB | IGFBP6 | PDPN | SRPX2 | LCP1 |
| CHI3L1 | IL15RA | PGCP | ST14 | PROCR |
| CLCF1 | IL1R1 | PHF11 | STAB1 | PTGER4 |
| CLEC2B | IL4R | PIGP | STAT6 | PTPN22 |
| CLIC1 | IQGAP1 | PLAU | STXBP2 | PTPN6 |
| CNN2 | ITGA4 | PLAUR | SWAP70 | PTPRC |
| COL1A1 | ITGA5 | PLK3 | SYNGR2 | TEC |
| COL1A2 | ITGAM | PLS3 | SYPL1 | TES |
| COL5A1 | ITGB2 | POLD4 | TCIRG1 | TGFBI |
|  |  |  | TGOLN2 | TGFBR2 |
